# Supplementary material for: Opposing roles for striatonigral and striatopallidal neurons in dorsolateral striatum in consolidating new instrumental actions
Source: Nat Commun. 2021 Aug 25;12:5121. doi: 10.1038/s41467-021-25460-3 (PMC8387469; doi:10.1038/s41467-021-25460-3)
Supplement: Supplementary file 1 — Supplementary Information [file 41467_2021_25460_MOESM1_ESM.pdf]

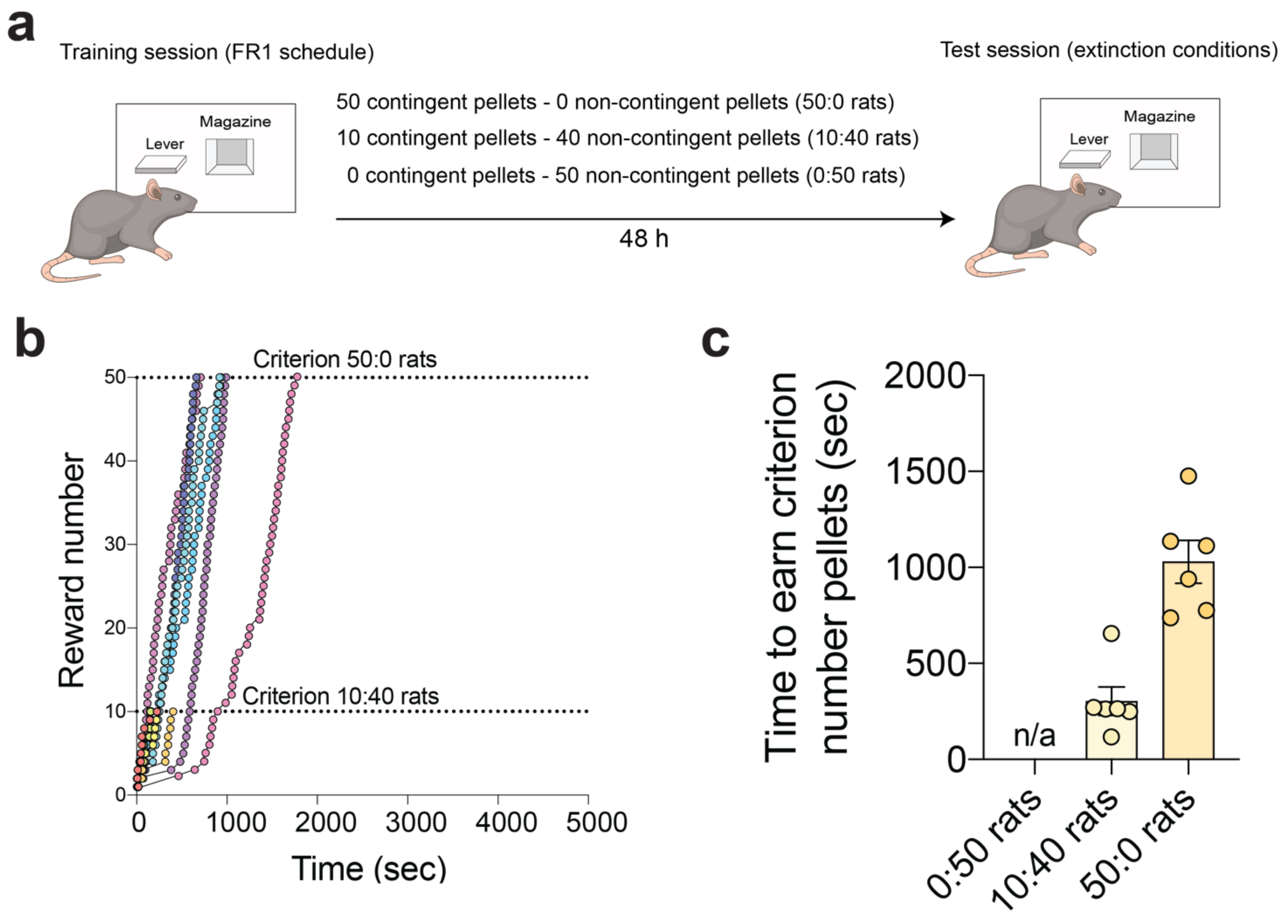

Supplementary Figure 1. Within-session training profiles of 10:40 and 50:0 rats.
a, Graphical representation of task used to investigate mechanisms of new instrumental conditioning. **b,** Event records from individual 10:40 and 50:0 rats as they acquired a new lever press response for food pellet rewards. **c,** Mean (±s.e.m.) time (sec) required for 10:40 and 50:0 rats to earn their criterion number of pellets during the acquisition session.

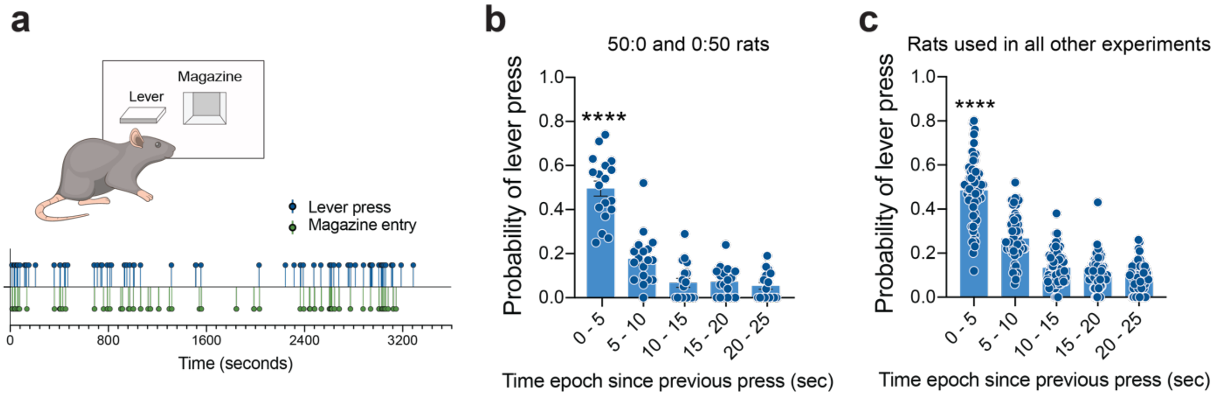

Supplementary Figure 2. Defining bouts of responding.
a, Representative event record from 50:0 rat showing lever presses and magazine entries during retention test. Note the frequent clustering of lever presses into bouts of responding. **b,** The probability that a lever-press would occur within given time intervals (5 sec epochs) after emission of a previous lever-press response was calculated for 0:50, 10:40 and 50:0 rats during the retention test. Probability that a subsequent lever-press response would occur was maximal within 5 sec of a preceding response. Consequently, a bout was defined as any two or more lever-press responses that occurred within 5 sec; $F_{(4,85)}$=62.16, p<0.0001; ****P<0.0001 compared with each of the other epochs. **c,** The same probability that a lever-press response would occur within time epochs after a preceding lever-press was calculated during retention tests for all control rats used in every experiment. Probability was again maximal within 5 sec of a preceding response; $F_{(4,295)}$=172.3, p<0.0001, One-way ANOVA; ****P<0.0001 compared with every other time epoch.

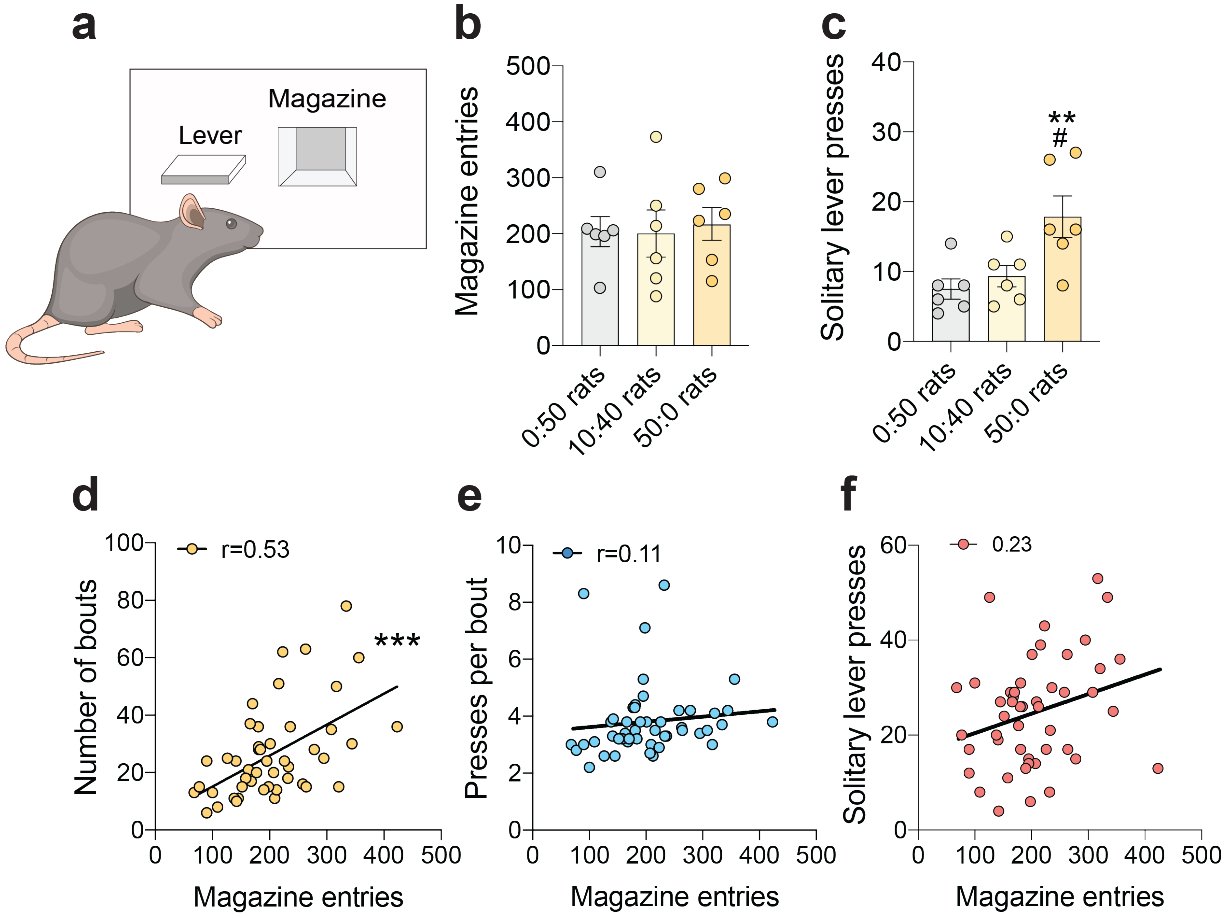

Supplementary Figure 3. Response bouts were linked to magazine entries.
a, Graphical representation of rat in the testing apparatus during the retention test when lever presses were recorded by had no scheduled consequences. **b**, Mean (±s.e.m.) number of magazine entries in 0:50 (*n*=6), 10:40 (*n*=6) and 50:0 (*n*=6) rats during the retention test; $F_{(2,15)}=0.07464$, $p=0.9284$, One-way ANOVA. **c**, Mean (±s.e.m.) number of solitary lever presses; $F_{(2,15)}=6.811$, $p=0.0079$, One-way ANOVA; **$p=0.0092$ compared with 0:50 rats, #$p=0.0311$ compared with 0:50 rats, post-hoc test. **d**, Numbers of bouts of responding for each 0:50, 10:40 and 50:0 rat was correlated with their number of magazine entries. Pearson r = 0.5299, ***$p<0.0001$. **e**, Numbers of presses per bout of responding for each 0:50, 10:40 and 50:0 rat was correlated with their number of magazine entries. Pearson r=0.1125, $p=0.4465$. **f**, Numbers of solitary lever presses for each 0:50, 10:40 and 50:0 rat was correlated with their number of magazine entries. Pearson r=0.2799, $p=0.0540$.

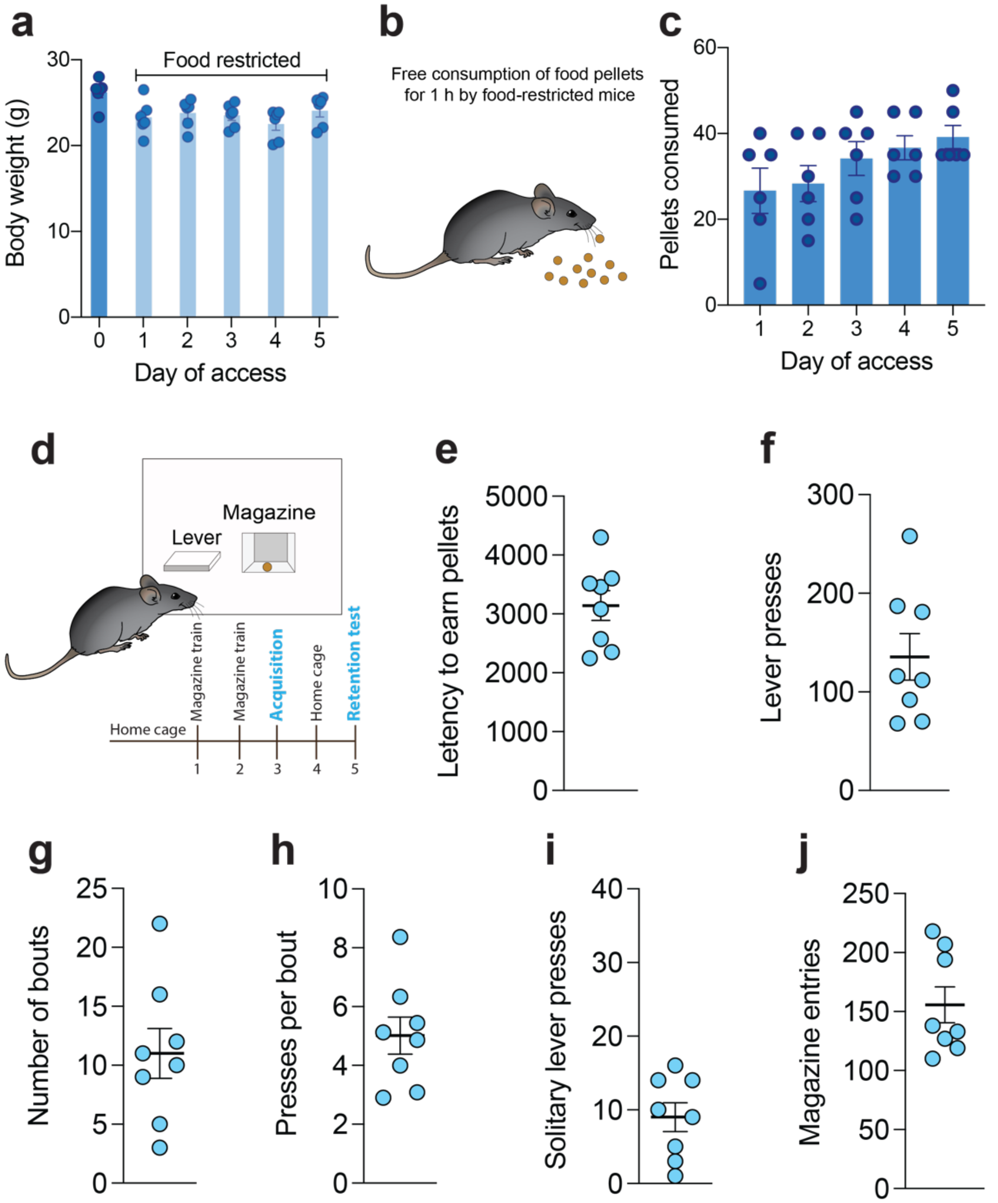

Supplementary Figure 4. Single session acquisition of new instrumental action in mice.
a, Mean (±s.e.m.) daily body weights of a group male C57BL6/J mice (*n*=6) at baseline (day 0) and during 5 days of food restriction. Food restriction decreased body weights in these animals as expected; $F_{(5,25)}$=28.34, p<0.0001. **b**, Graphical representation of mouse consuming chow pellets. **c**, Mean (±s.e.m.) number of chow pellets consumed when mice were provided unlimited access to pellets for 60 min each day in their home-cage at baseline and during 5 days of food restriction. Mice consumed on average no more than 40 pellets. Consequently, we limited the maximal number of pellets that hungry mice could earn during an acquisition session to 30 pellets. **d**, Graphical representation of mouse in the testing apparatus during the retention test when lever presses were recorded by had no scheduled consequences. **e**, Mean (±s.e.m.) latency (sec) required for mice to earn criterion number of food pellets during acquisition session under a FR1 schedule. **f**, Mean number of lever-presses (±s.e.m) during retention session. **g**, Mean number of response bouts (±s.e.m). **h**, Mean number of lever presses per response bout (±s.e.m). **i**, Mean number of solitary lever presses (±s.e.m). **j**, Mean number of magazine entries (±s.e.m).

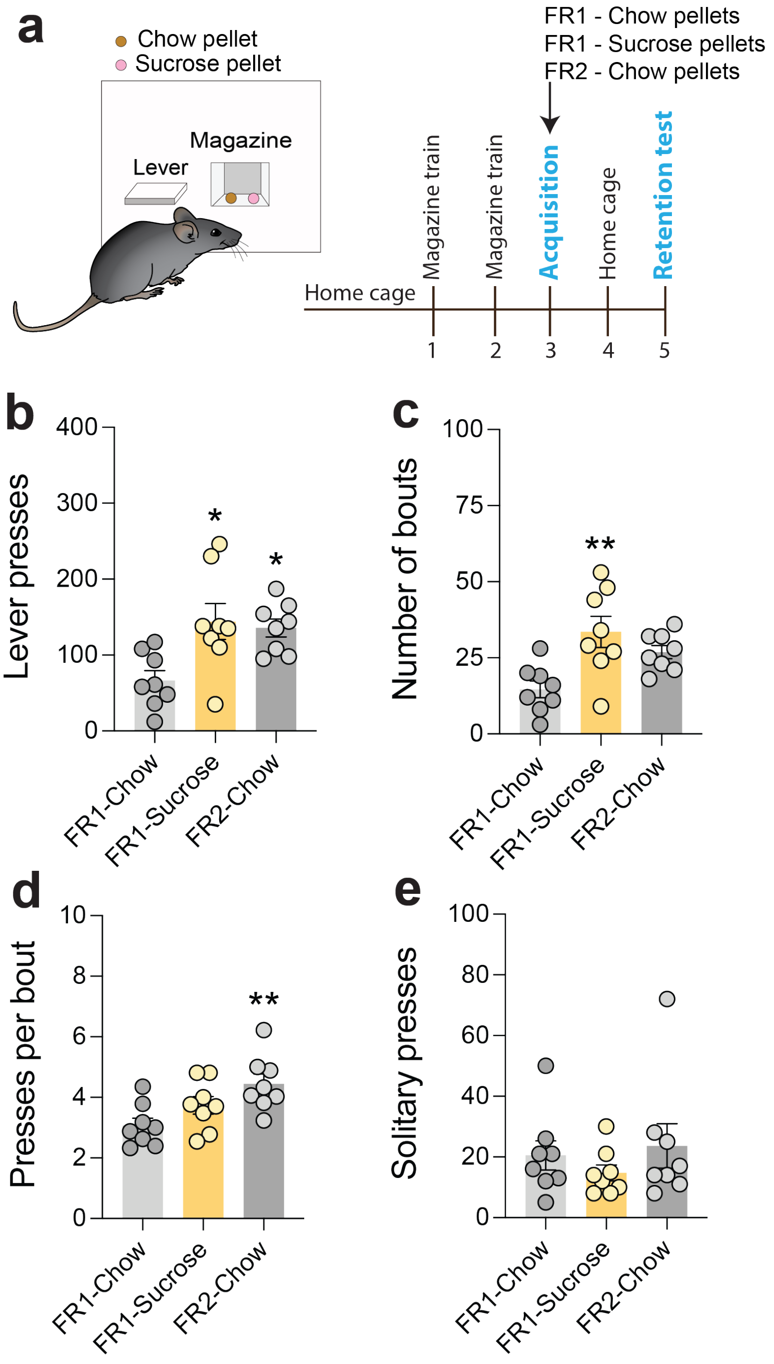

Supplementary Figure 5. Contingencies during training alter bout structure in retention test. **a**, Graphical representation of task used to investigate the effects of manipulating reinforcer magnitude and effort required to earn a reinforcer during training on the structure of response bouts during the retention test. **b**, Mean (±s.e.m.) number of lever presses emitted by FR1-Chow, FR1-Sucrose and FR2-Chow mice during retention test; $F_{(2, 21)}$=6.275, *p*=0.0073; **P*<0.05 compared with FR1-Chow mice. **c**, Mean (±s.e.m.) number of response bouts; $F_{(2, 21)}$=7.163, *p*=0.0042; ***P*<0.01 compared with FR1-Chow mice. **d**, Mean (±s.e.m.) number of lever-presses per response bout; F($F_{(2, 21)}$=5.713, *p*=0.0104; ***P*<0.01 compared with FR1-Chow mice. **e**, Mean (±s.e.m.) number of solitary lever presses.

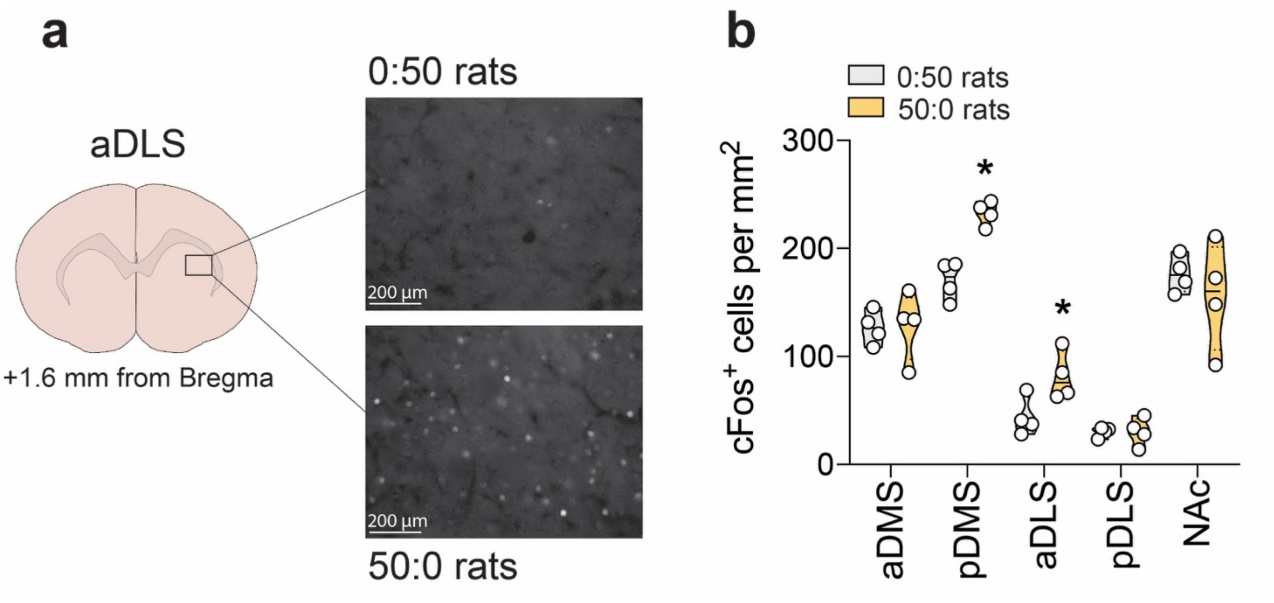

Supplementary Figure 6. c-Fos+ cells in striatum of 0:50 and 50:0 rats.
a, Representative image of c-Fos immunoreactivity in the aDLS of 0:50 (*n*=4) and 50:0 (*n*=4) rats.
b, Mean (±s.e.m) number of c-Fos immunoreactive cells in aDMS, pDMS, aDLS, pDMS and NAc of 0:50 and 50:0 rats. **P*>0.05 compared with 0:50 rats, unpaired two-tailed t-test.

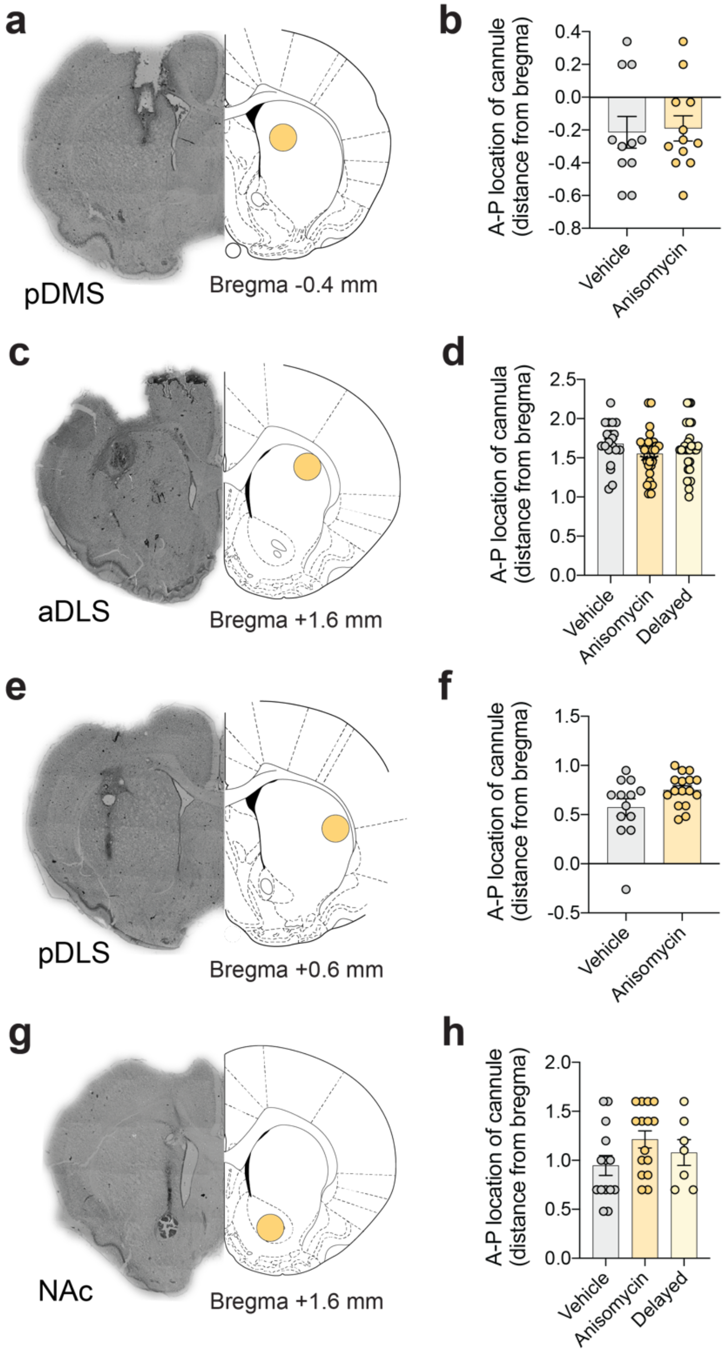

Supplementary Figure 7. Location of anisomycin injections in striatum of rats.

a, Representative image of cannula placement in pDMS of rats. **b**, Location of injector tip targeted to the pDMS in the anterior-posterior (A-P) plane (mm from Bregma), according to the rat brain atlas of Paxinos and Watson (2013) for each rat injected with vehicle or anisomycin. **c**, Representative image of cannula placement in aDLS of rats. **d**, Location of injector tip targeted to the aDLS in the A-P plane (mm from Bregma) for each rat injected with vehicle or anisomycin. **e**, Representative image of cannula placement in pDLS of rats. **f**, Location of injector tip targeted to the pDLS in the A-P plane (mm from Bregma) for each rat injected with vehicle or anisomycin. **g**, Representative image of cannula placement in NAc of rats. **h**, Location of injector tip targeted to the NAc in the A-P plane (mm from Bregma) for each rat injected with vehicle or anisomycin.

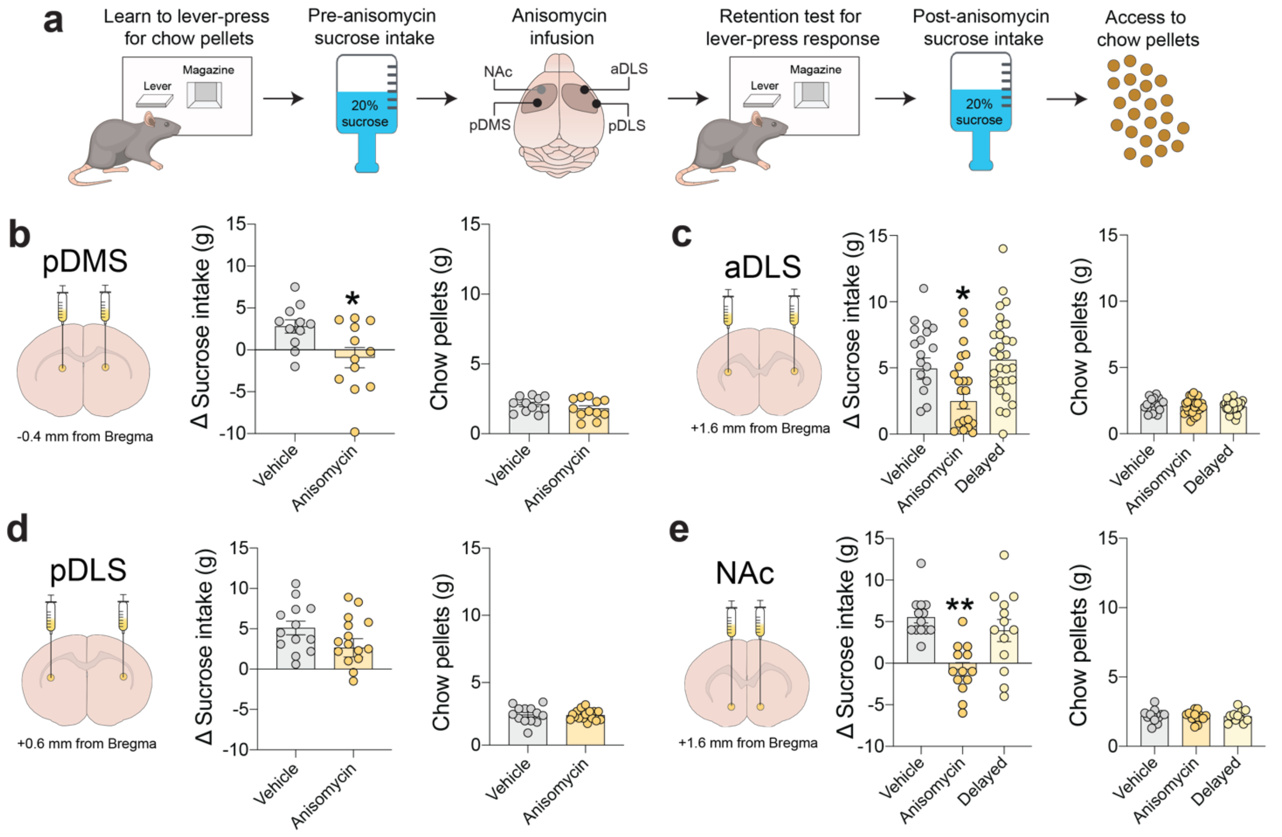

Supplementary Figure 8. Protection of chow pellet valuation.

a, Graphical representation of experimental design. **b**, Graphical representation of coronal image of rat brain showing injection of vehicle or anisomycin to pDMS (left). Change (g) (±s.e.m.) in post-injection consumption compared with pre-injection levels of sucrose solution (20% w/v) (middle) or chow pellets (right) in vehicle-injected (*n*=12) and anisomycin-injected (*n*=12) rats; $*P_{(t=2.526, df=21)}=0.0196$ compared with vehicle-injected rats, unpaired two-tailed t-test. **c**, Graphical representation of coronal image of rat brain showing injection of vehicle or anisomycin to the aDLS (left). Change (g) (±s.e.m.) in post-injection consumption compared with pre-injection levels of sucrose solution (middle) or chow pellets (right) in vehicle-injected (*n*=20), anisomycin-injected (*n*=28) or anisomycin-injected after 6 h delay (*n*=30) rats (right); $F_{(2,74)}=6.67$, $p=0.0022$, $*P=0.0292$, compared with vehicle-injected rats. **d**, Graphical representation of coronal image of rat brain showing injection of vehicle or anisomycin to the pDLS (left). Change (g) (±s.e.m.) in post-injection consumption compared with pre-injection levels of sucrose solution (middle) or chow pellets (right) in vehicle-injected (*n*=13) and anisomycin-injected (*n*=16) rats (right). **e**, Graphical representation of coronal image of rat brain showing injection of vehicle or anisomycin to the NAc (left). Change (g) (±s.e.m.) in post-injection consumption compared with pre-injection levels of sucrose solution (middle) or chow pellets (right) in vehicle-injected (*n*=13), anisomycin-injected (*n*=13) or anisomycin-injected after 6 h delay (*n*=13) rats (right); $F_{(2,36)}=11.01$, $p<0.0001$, $**P=0.0292$, compared with vehicle-injected rats.

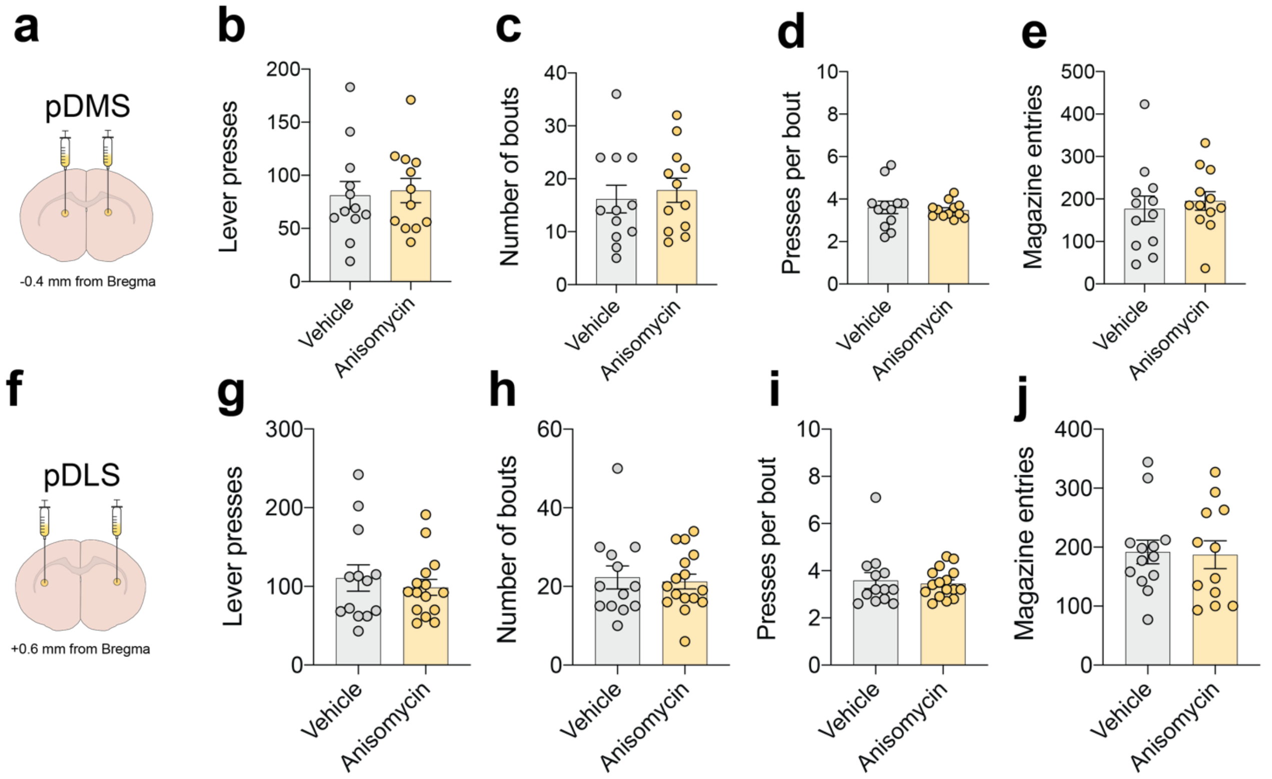

Supplementary Figure 9. Protein synthesis in pDMS or pDLS not required for consolidation. **a,** Graphical representation of coronal image of rat brain depicting the injection of vehicle or anisomycin into pDMS. **b**, Mean (±s.e.m.) number of lever presses in vehicle-treated rats (*n*=12) and anisomycin-treated rats (*n*=12); $P_{(t=2.562,\ df=22)}$=0.8002, unpaired two-tailed t-test. **c,** Mean (±s.e.m.) number of bouts of lever presses; $P_{(t=0.4785,\ df=22)}$=0.6370. **d,** Mean number of responses per bout (±s.e.m.); $P_{(t=0.3949,\ df=22)}$=0.6967. **e,** Mean (±s.e.m.) number of magazine entries; $P_{(t=0.5003,\ df=22)}$=0.6218. **f,** Graphical representation of coronal image of rat brain depicting the injection of vehicle or anisomycin into pDLS. **g**, Mean (±s.e.m.) number of lever presses in vehicle-treated rats (*n*=13) and anisomycin-treated rats (*n*=15); $P_{(t=0.628,\ df=27)}$=0.5355, unpaired two-tailed t-test. **h,** Mean (±s.e.m.) number of bouts of lever presses; $P_{(t=0.3143,\ df=27)}$=0.7557. **i,** Mean number of responses per bout (±s.e.m.); $P_{(t=0.3704,\ df=27)}$=0.7140. **j,** Mean (±s.e.m.) number of magazine entries; $P_{(t=0.1538,\ df=27)}$=0.8791.

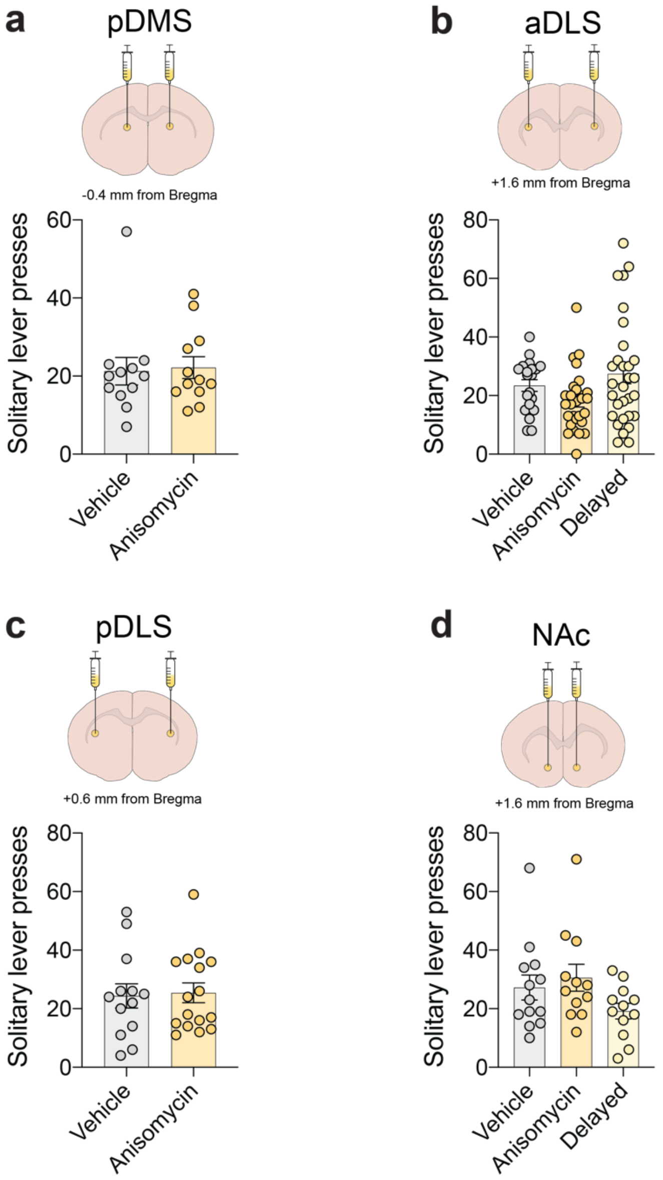

Supplementary Figure 10. Solitary responses during retention test in anisomycin rats.
a, Graphical representation of coronal image of rat brain showing injection of vehicle or anisomycin to pDMS (upper). Mean (±s.e.m.) number of solitary lever press responses in vehicle-injected (*n*=12) and anisomycin-injected (*n*=12) rats (lower); $P_{(t=0.2034,\ df=22)}$=0.8407, unpaired two-tailed t-test. **b**, Graphical representation of coronal image of rat brain showing injection of vehicle or anisomycin to the aDLS (upper). Mean (±s.e.m.) number of solitary lever press responses in vehicle-injected (*n*=20), anisomycin-injected (*n*=28) or anisomycin-injected after 6 h delay (*n*=30) rats (lower); $F_{(2,\ 75)}$=3.37, *p*=0.0397, One-way ANOVA; *p*=0.0306 anisomycin-treated compared with delayed group. **c**, Graphical representation of coronal image of rat brain showing injection of vehicle or anisomycin to pDLS (upper). Mean (±s.e.m.) number of solitary lever press responses in vehicle-injected (*n*=13) and anisomycin-injected (*n*=16) rats (lower); $P_{(t=0.1994,\ df=27)}$=0.8434, unpaired two-tailed t-test. **d**, Graphical representation of coronal image of rat brain showing injection of vehicle or anisomycin to the NAc (upper). Mean (±s.e.m.) number of solitary lever press responses in vehicle-injected (*n*=13), anisomycin-injected (*n*=13) or anisomycin-injected after 6 h delay (*n*=13) rats (lower); $F_{(2,\ 34)}$=2.15, *p*=0.1321, One-way ANOVA.

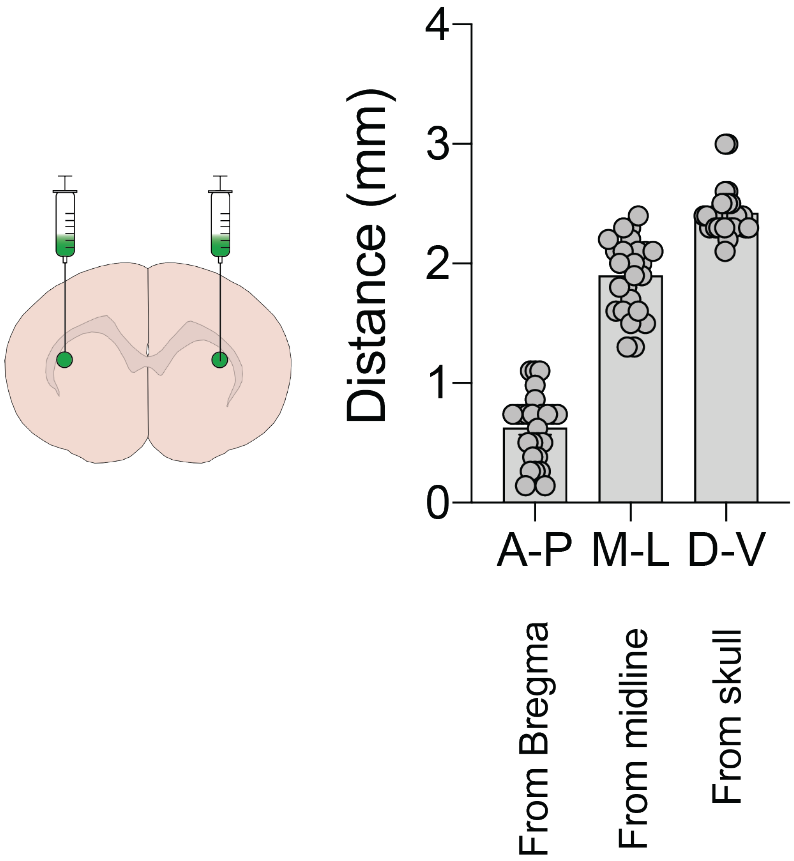

Supplementary Figure 11. Location of virus injections in aDLS of mice.
a, Graphical representation of AAV-hM4Di-GFP virus injected site in aDLS of mice. **b**, Mean (±s.e.m.) and individual data for the location of virus-derived fluorescence in striatum given in stereotaxic coordinates according to the mouse brain atlas of Paxinos and Franklin (2019) in the anterior-posterior (A-P; mm from Bregma), medial-lateral (M-L; mm from midline) and dorsal-ventral (D-V; mm from skull) planes for each mouse used in the study.

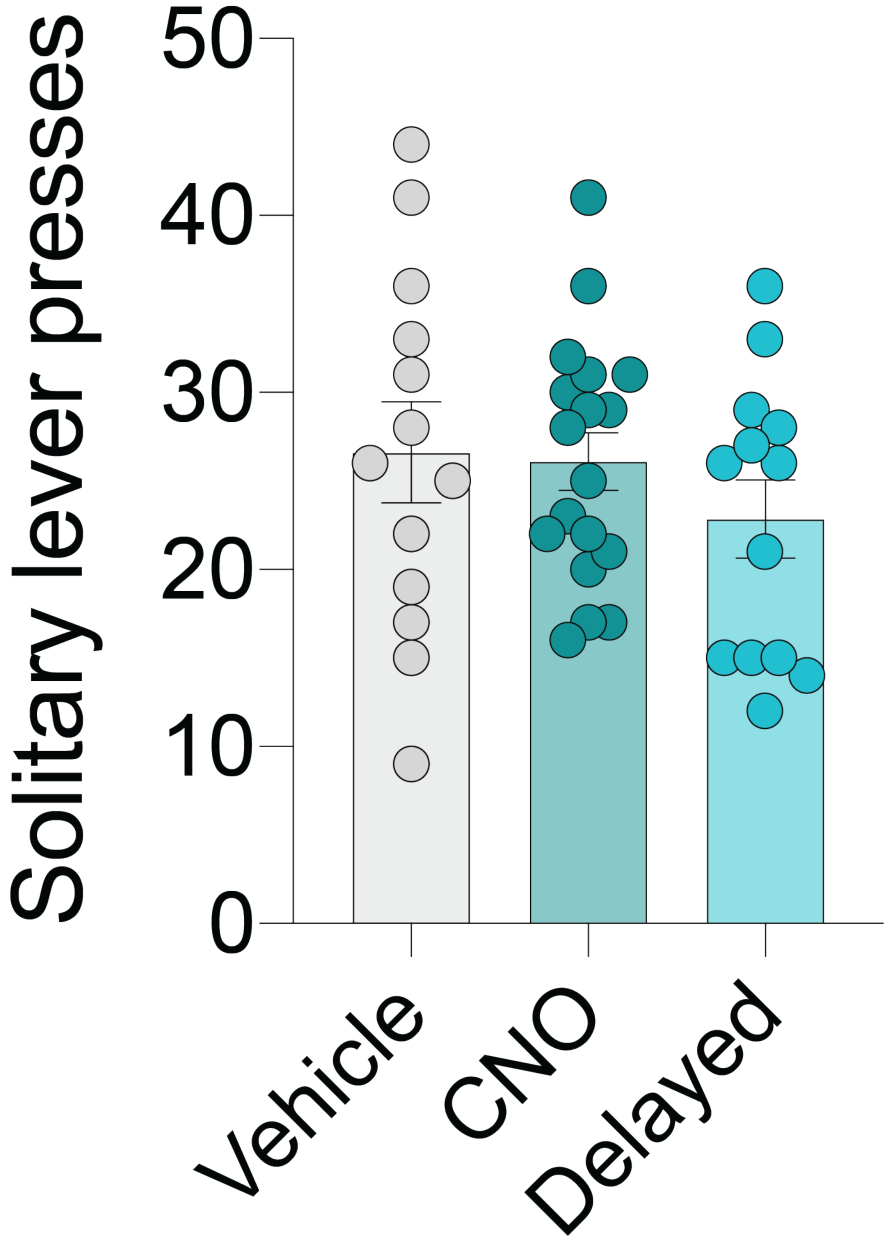

Supplementary Figure 12. Solitary responses during retention test in DREADD mice
Mean (±s.e.m.) number of solitary lever press responses in vehicle-injected (*n*=13), CNO-injected (*n*=18) mice or CNO-injected after 6 h delay (*n*=13) mice; $F_{(2,41)}=0.8087$, $p=0.4524$; One-way ANOVA.

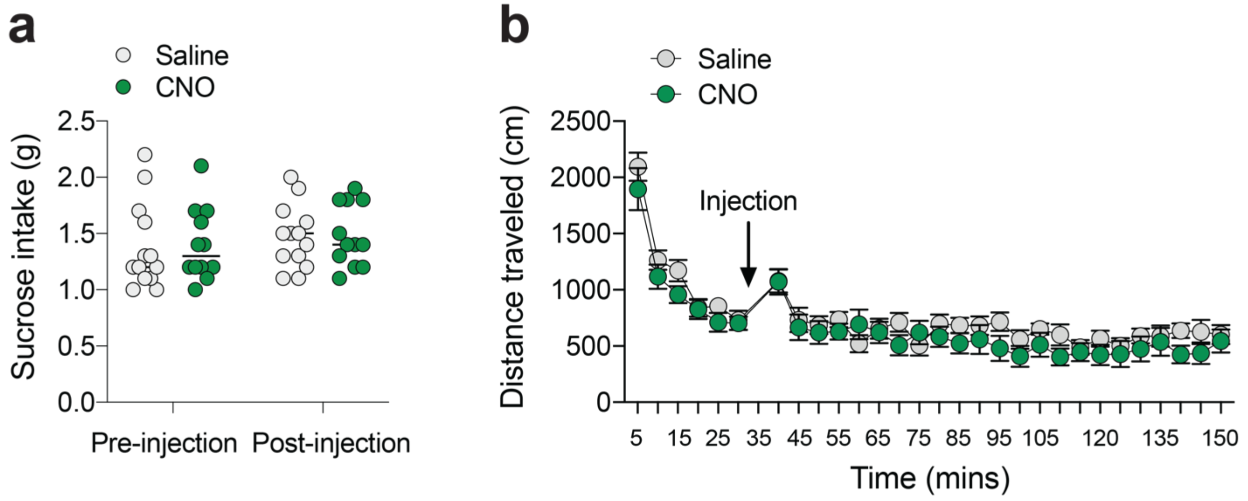

Supplementary Figure 13. Sucrose intake and locomotion in DREADD mice.
a, Mean (±s.e.m.) consumption of sucrose solution (20% w/v) before and after vehicle or CNO injection in mice (*n*=13) injected into the aDLS with AAV-hM4Di-GFP treated with vehicle or CNO; $F_{(1,23)}$=0.003069, *p*=0.9563, interaction in Two-way repeated measures ANOVA. **b**, Mean (±s.e.m.) distance travelled (cm) in 5 min epochs in mice (*n*=13) injected into the aDLS with AAV-hM4Di-GFP treated with vehicle or CNO; $F_{(28,307)}$=0.9845, interaction in Two-way repeated measures ANOVA.

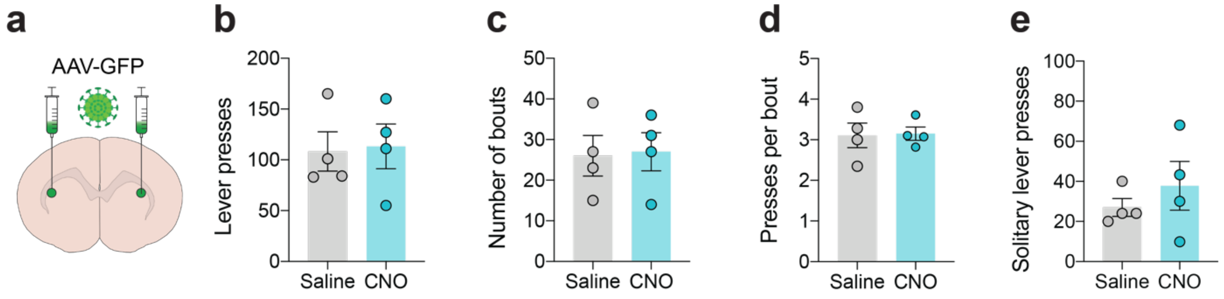

Supplementary Figure 14. CNO had no effects on consolidation in AAV-GFP mice.
a, Graphical representation of coronal image of mouse brain depicting injection site of AAV-GFP in aDLS. **b**, Mean (±s.e.m.) number of lever presses in vehicle-treated (*n*=4) and CNO-treated mice (*n*=4) mice; $P_{(t=0.1709,\ df=6)}$=0.8699, unpaired two-tailed t-test. **c,** Mean (±s.e.m.) number of bouts of lever presses; $P_{(t=0.1456,\ df=6)}$=0.8890. **d,** Mean (±s.e.m.) number of responses per bout; $P_{(t=0.1237,\ df=6)}$=0.9056. **e,** Mean (±s.e.m.) number of solitary lever presses; $P_{(t=0.8332,\ df=6)}$=0.4366.

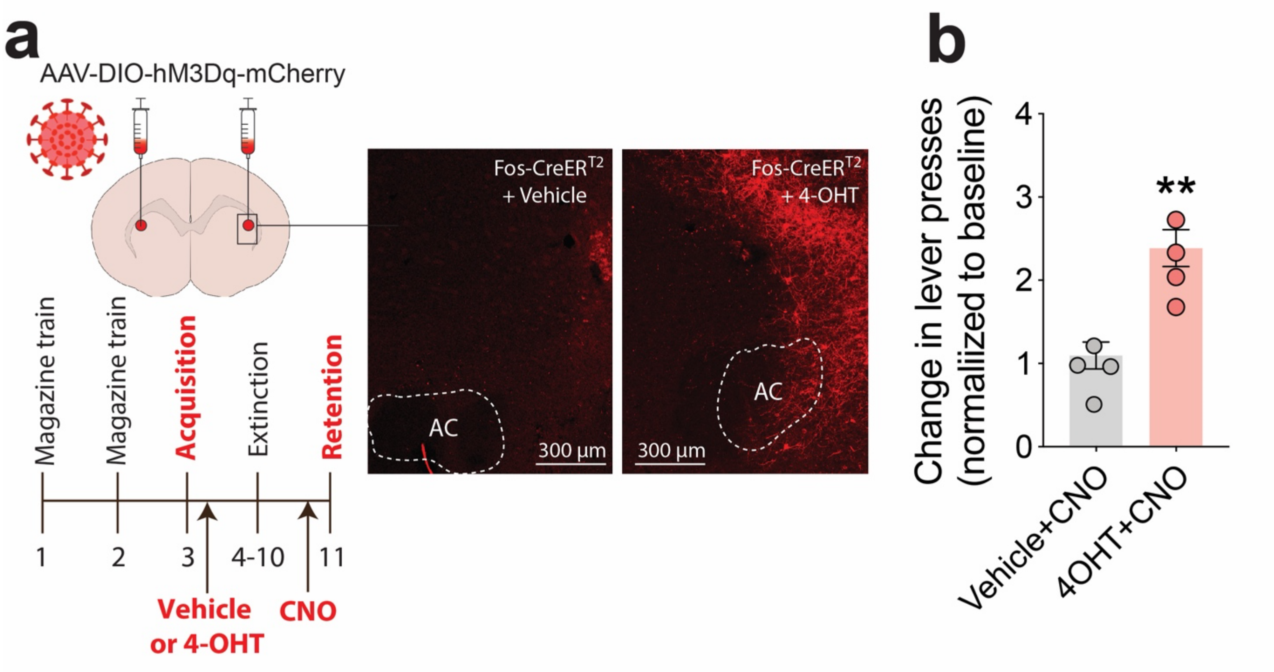

Figure 15. Chemogenetic stimulation of aDLS ensembles facilitates new learning.
a, Graphical representation of excitatory hM3Di-mCitrine DREADD virus delivered to aDLS of mice, experimental design, and representative mCitrine image from aDLS-injected mouse. **b**, Change in mean (±s.e.m.) number of lever presses in response to treatment (normalized to baseline) by CNO treatment during a retention test in *FosCre*^{ERT2} mice previously treated with vehicle or 4-OHT immediately after acquisition of a new lever-press response during a single training session; ***P*(t=4.696,df=6)=0.0033, unpaired two-tailed t-test.

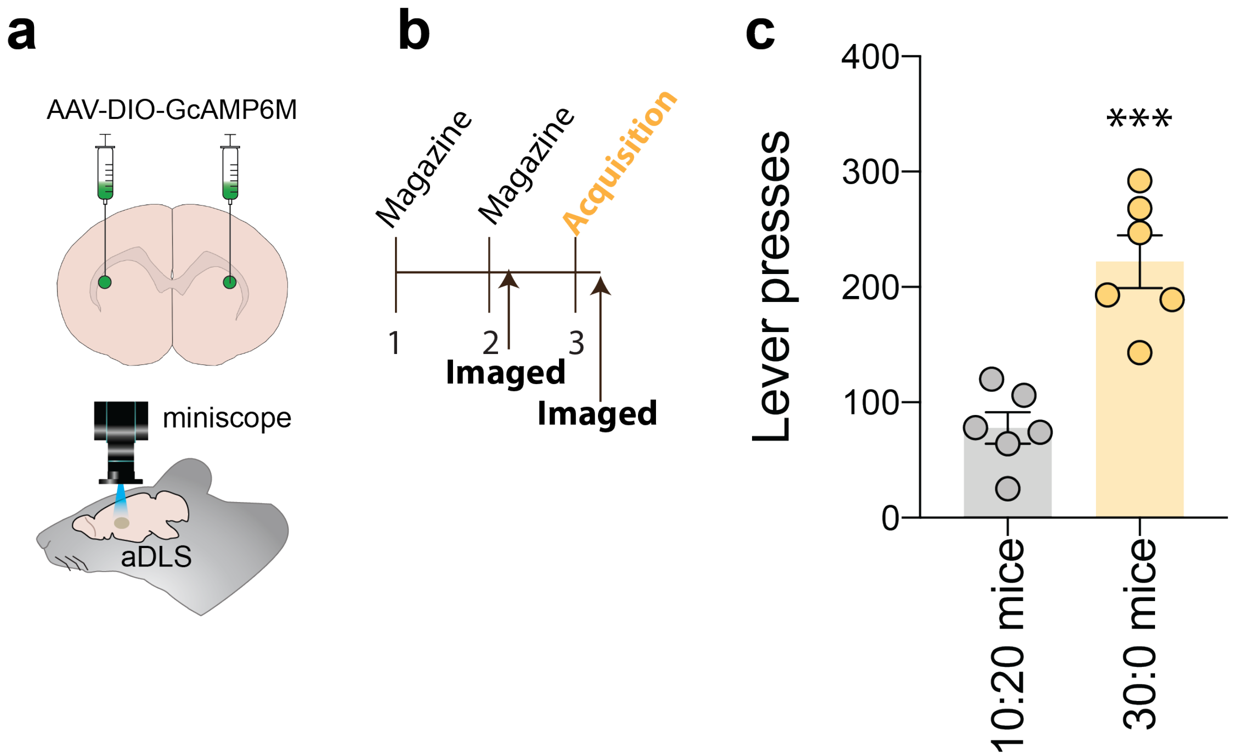

Supplementary Figure 16. Lever-presses during retention test in 10:20 and 30:0 mice.
a, Rendering of coronal image of mouse brain depicting injection site of AAV-GCaMP6M and site of GRINS lens implantation in the aDLS of D1-Cre (*n*=6) and D2-Cre mice (*n*=6) that were allocated to 10:20 or 30:0 groups (above) and representation of a miniscope attached to head a mouse to collect calcium events from D1 and D2-MSNs. **b**, Graphical representation of experimental design. **c**, Mean (±s.e.m.) number of lever presses in 10:20 (*n*=6; D1-Cre and D2-Cre mice collapsed) and 10:20 (*n*=6; D1-Cre and D2-Cre mice collapsed) mice during retention test; $^{***}P_{(t=5.4,\ df=10)}=0.0003$, unpaired two-tailed t-test.

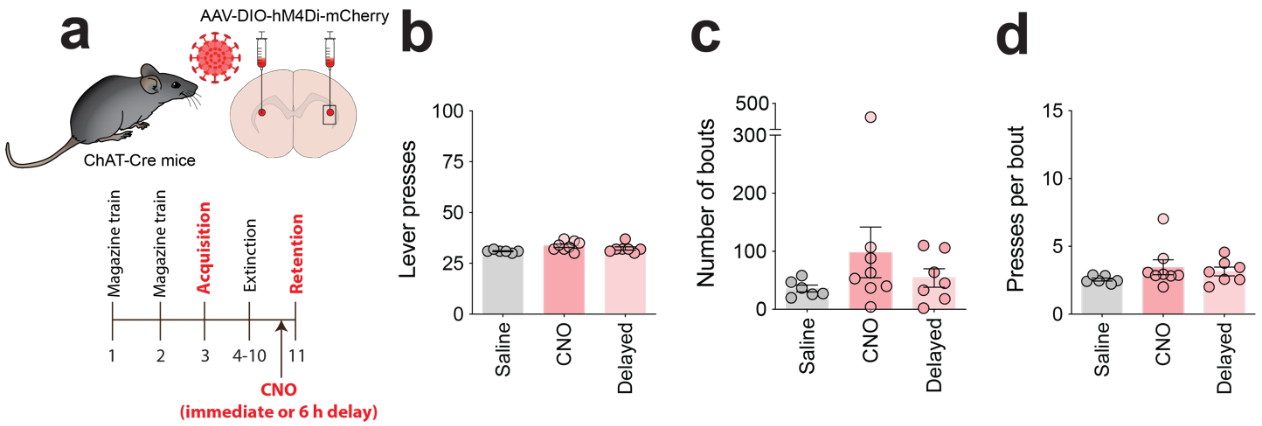

Supplementary Figure 17. Cholinergic interneurons in aDLS do not regulate consolidation. **a,** Graphical representation of inhibitory hM4Di-mCherry DREADD virus delivered to aDLS of ChAT-Cre mice and experimental design. **b**, Mean (±s.e.m.) number of lever presses in vehicle-treated ChAT-Cre mice (*n*=6), ChAT-Cre mice infused with CNO after new learning (*n*=8), and ChAT-Cre mice infused with CNO 6 h after new learning (*n*=7); $F_{(2,18)}=3.096$, $p=0.0699$, One-way ANOVA. **c,** Mean (±s.e.m.) number of bouts of lever presses; $F_{(2,18)}=1.123$, $p=0.3470$. **d,** Mean (±s.e.m.) number of lever presses per bout; $F_{(2,18)}=1.186$, $p=0.3283$.

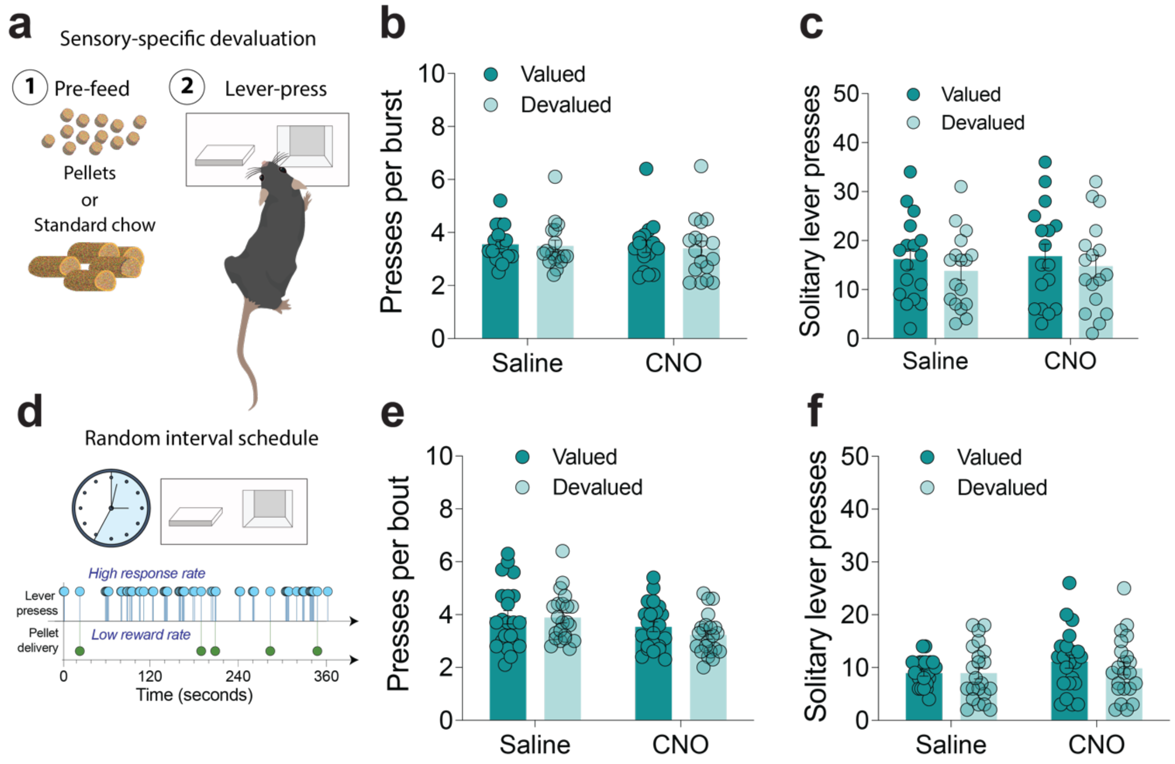

Supplementary Figure 18. Bout density and solitary response after aDLS inactivation in valued and devalued conditions under FR1 and RI60 reinforcement schedules.

a, Graphical representation of the sensory-specific satiety-induced devaluation procedure used to assess the goal-directedness of lever-pressing behavior during the retention test in mice. Mice were pre-fed with chow (valued condition) or pellets (devalued condition) (1), then permitted to lever-press under extinction conditions (2). **b**, Mean (±s.e.m.) numbers of lever presses per bout under extinction conditions in mice expressing hM4Di in aDLS (*n*=22) trained under an FR1 schedule after vehicle or CNO injection in valued and devalued conditions; $F_{(1,21)}=0.8209$, $p=0.3752$; interaction effect in Two-way repeated-measures ANOVA. **c**, Mean (±s.e.m.) number of solitary lever presses; $F_{(1,21)}=0.6992$, $p=0.4125$**;** interaction effect in Two-way repeated-measures ANOVA. **d,** Graphical representation of the RI60 procedure used to assess habitual behavior (upper panel). Shown are data from a mouse lever-pressing under the RI60 schedule (first 360 sec from 3600 sec session), with characteristic high rates of responding and low rates of reward delivery. **e**, Mean (±s.e.m.) number of lever presses per bout under extinction conditions in mice expressing hM4Di in aDLS (*n*=22) trained under an RI60 schedule after vehicle or CNO injection in valued and devalued conditions; $F_{(1,16)}=0.01808$, $p=0.8947$, interaction effect in Two-way repeated-measures ANOVA. **f**, Mean (±s.e.m.) number of solitary lever presses; $F_{(1,16)}=0.01819$, $p=0.8944$.

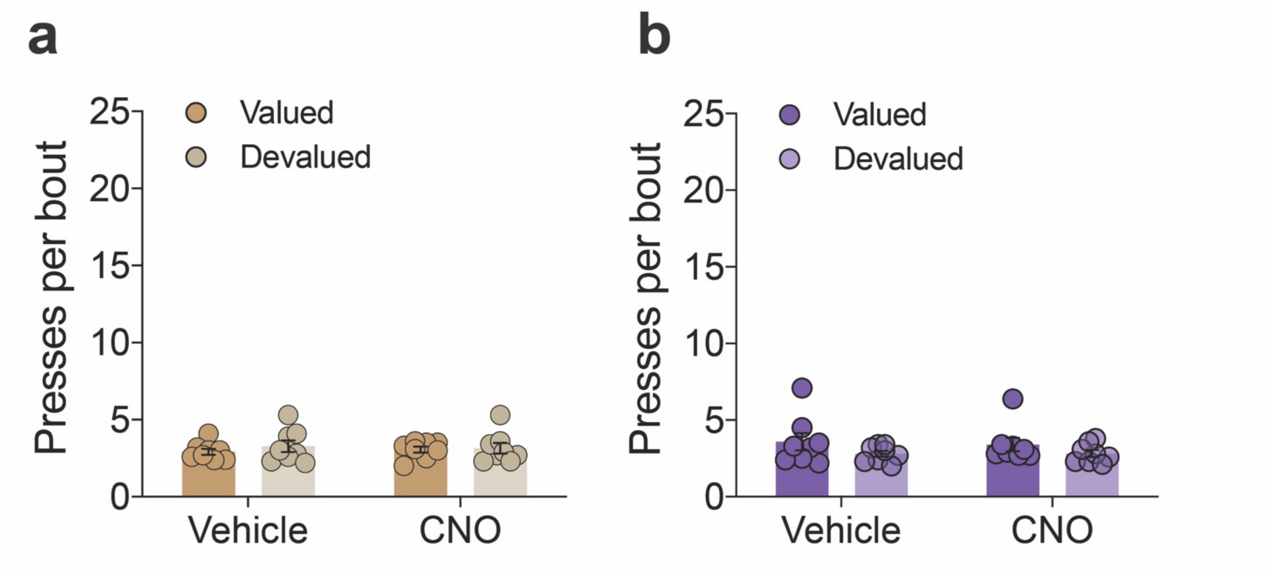

Supplementary Figure 19. Bout density in D1 and D2-cre mice in RI60 task.

a, Mean (±s.e.m.) numbers of presses per bout under extinction conditions during sensory-specific devaluation testing after RI60 training in D1-Cre mice expressing hM4Di in aDLS mice. $F_{(1,14)}$=0.3342, *p*=0.5724 interaction effect in Two-way repeated-measures ANOVA. **b,** Mean (±s.e.m.) numbers of presses per bout under extinction conditions during sensory-specific devaluation testing after RI60 training in D2-Cre mice expressing hM4Di in aDLS mice. $F_{(1,14)}$=0.07631, *p*=0.7864 interaction effect in Two-way repeated-measures ANOVA.

Supplemental Table 1: Abbreviations used in Figure 1:

aDLS – Anterior Dorsolateral Striatum
pDMS – Posterior Dorsomedial Striatum
CPu – Caudoputamen (Not otherwise specified)
NAc – Nucleus Accumbens
PD – Pallidum
DG – Dentate Gyrus
PrS – Presubiculum
PoS – Postsubiculum
Sub – Subiculum
CA1 – Field CA1
CA3 – Field CA3
Ent – Entorhinal Cortex
HPC – Hippocampus (Not otherwise specified)
RSC – Restrosplenial Cortex
ACC – Anterior Cingulate Cortex
PL – Prelimbic Cortex
IL – Infralimbic Cortex
PMA – Primary Motor Area
SMA – Secondary Motor Area
BMA – Basomedial Amygdala
BLA – Basolateral Amygdala
CeA – Central Amygdala
VTA – Ventral Tegmental Area

Supplemental Table 2: Primers Used.

**D1-Cre**	**Sequence:**
Primer1:	5′-GAACCTGATGGACATGTTCAGG-3′
Primer2:	5’-CGGCAAACGGACAGAAGCATT-3’

**D2-Cre**	**Sequence:**
Primer1:	5′-AGTGCGTTCGAACGCTAGAGCCTGT-3′
Primer2:	5’-CGGCAAACGGACAGAAGCATT-3’

**Chat-Cre**	**Sequence:**
Primer1:	5'-GCAAAGAGACCTCATCTGTGGA-3'
Primer2:	5'-GATAGGGGAGCAGCAACAAG-3'
Primer3:	5'-TTCACTGCATTCTAGTTGTGGT-3'

**FosCreERT2**	**Sequence:**
Primer1:	5’-CACCAGTGTCTACCCCTGGA-3’
Primer2:	5’-CGGCTACACAAAGCCAAACT-3’
Primer3:	5’ CGCGCCTGAAGATATAGAAGA-3’

**Fos-2AiCreER**	**Sequence:**
Primer1:	5'-GTCCGGTTCCTTCTATGCAG-3'
Primer2:	5'-GAACCTTCGAGGGAAGACG-3'
Primer3:	5'-CCTTGCAAAAGTATTACATCACG-3'
