# Supplementary material for: Opposing roles for striatonigral and striatopallidal neurons in dorsolateral striatum in consolidating new instrumental actions
Source: Nat Commun. 2021 Aug 25;12:5121. doi: 10.1038/s41467-021-25460-3 (PMC8387469; doi:10.1038/s41467-021-25460-3)
Supplement: Supplementary file 3 — Reporting Summary [file 41467_2021_25460_MOESM3_ESM.pdf]

nature research

Corresponding author(s): Paul J. Kenny, PhD

Last updated by author(s): 7/5/2021

Reporting Summary

Nature Research wishes to improve the reproducibility of the work that we publish. This form provides structure for consistency and transparency in reporting. For further information on Nature Research policies, see our Editorial Policies and the Editorial Policy Checklist.

Statistics

For all statistical analyses, confirm that the following items are present in the figure legend, table legend, main text, or Methods section.

n/a	Confirmed	
☐	☒	The exact sample size (*n*) for each experimental group/condition, given as a discrete number and unit of measurement
☐	☒	A statement on whether measurements were taken from distinct samples or whether the same sample was measured repeatedly
☐	☒	The statistical test(s) used AND whether they are one- or two-sided<br>*Only common tests should be described solely by name; describe more complex techniques in the Methods section.*
☐	☒	A description of all covariates tested
☐	☒	A description of any assumptions or corrections, such as tests of normality and adjustment for multiple comparisons
☐	☒	A full description of the statistical parameters including central tendency (e.g. means) or other basic estimates (e.g. regression coefficient) AND variation (e.g. standard deviation) or associated estimates of uncertainty (e.g. confidence intervals)
☐	☒	For null hypothesis testing, the test statistic (e.g. *F*, *t*, *r*) with confidence intervals, effect sizes, degrees of freedom and *P* value noted<br>*Give P values as exact values whenever suitable.*
☒	☐	For Bayesian analysis, information on the choice of priors and Markov chain Monte Carlo settings
☒	☐	For hierarchical and complex designs, identification of the appropriate level for tests and full reporting of outcomes
☐	☒	Estimates of effect sizes (e.g. Cohen's *d*, Pearson's *r*), indicating how they were calculated

Our web collection on statistics for biologists contains articles on many of the points above.

Software and code

Policy information about availability of computer code

Data collection	Calcium imaging data was acquired using open-source miniscopes (miniscope.org) V3, additional information on data acquisition can be found at http://miniscope.org.
Data analysis	Calcium imaging and light-sheet microscopy data were analyzed on Ubuntu 18.04LTS, and ImageJ version 1.53c was used for image visualization. Calcium imaging data was analyzed via MATLAB scripts adapted from Piatkevich et al., PMID: 31597963, and Gritton et al., PMID: 30804530) and used on MATLAB version R2017a. Scripts are available from GitHub at: http://www.github.com/KennyLabSinai/SmithJonkmanNatComm2021. Whole-brain c-Fos imaging was analyzed using Python version 2.7, ClearMap version 1.0 (www.github.com/ChristophKirst/ClearMap), as well as custom scripts for quantifying cell counts in striatal subregions (www.github.com/alexcwsmith/imageProcessing). Statistics were run using SciPy version 1.4.1 and SciKit-Learn version 0.22.2. Visualization of tSNE plots used Matplotlib version 3.2.1 and Seaborn v0.10.0.0. For all experiments other than calcium imaging and whole-brain Fos mapping, data was analyzed with GraphPad Prism version 7+.

For manuscripts utilizing custom algorithms or software that are central to the research but not yet described in published literature, software must be made available to editors and reviewers. We strongly encourage code deposition in a community repository (e.g. GitHub). See the Nature Research guidelines for submitting code & software for further information.

Data

Policy information about availability of data

All manuscripts must include a data availability statement. This statement should provide the following information, where applicable:

- Accession codes, unique identifiers, or web links for publicly available datasets
- A list of figures that have associated raw data
- A description of any restrictions on data availability

Raw data will be made available upon request.

Field-specific reporting

Please select the one below that is the best fit for your research. If you are not sure, read the appropriate sections before making your selection.

[x] Life sciences [ ] Behavioural & social sciences [ ] Ecological, evolutionary & environmental sciences

For a reference copy of the document with all sections, see nature.com/documents/nr-reporting-summary-flat.pdf

Life sciences study design

All studies must disclose on these points even when the disclosure is negative.

Sample size	Sample sizes were chosen based on analysis of statistical power (using G*Power 3.1.9.6) from previous experiments with similar design in our laboratory.
Data exclusions	Exclusion criteria for these experiments was placement of microinjection cannulae or virus expression, and only animals that had misplaced targeting of microinjections were excluded.
Replication	Main findings in rats were replicated in 203 independent cohorts. Non-Cre-dependent DREADD experiments in mice were replicated in two independent cohorts. Presented data represent the combined cohorts. Experiments involving Cre-driver mice (D1-Cre, D2-Cre and ChAT-Cre) were performed in only single cohorts. All attempts a replication were successful.
Randomization	Rigorous counterbalancing procedures were used to avoid any effects of cage mates, and prior behavioral and pharmacological history. As outlined in the Methods section, counterbalancing for post-acquisition treatment with anisoymycin or CNO was based on latency to meet acquisition criteria, and counterbalancing for devaluation experiments was based on lever-pressing during FR/VI schedules, also ensuring that no cage had all animals in the same treatment group.
Blinding	Experimenters were blinded to injection conditions for behavioral experiments. All imaging and computational analysis was conducted by blinded investigators. For contingency management experiments, it was not always possible to blind experimenters, due to the need to pre-feed with chow vs. 20mg food pellets, and to pair yoked animals to operant responding animals (e.g. 10:50 rats yoked to 50:0 rats).

Reporting for specific materials, systems and methods

We require information from authors about some types of materials, experimental systems and methods used in many studies. Here, indicate whether each material, system or method listed is relevant to your study. If you are not sure if a list item applies to your research, read the appropriate section before selecting a response.

Materials & experimental systems

n/a	Involved in the study	
[ ]	[x]	Antibodies
[x]	[ ]	Eukaryotic cell lines
[x]	[ ]	Palaeontology and archaeology
[ ]	[x]	Animals and other organisms
[x]	[ ]	Human research participants
[x]	[ ]	Clinical data
[x]	[ ]	Dual use research of concern

Methods

n/a	Involved in the study	
[x]	[ ]	ChIP-seq
[x]	[ ]	Flow cytometry
[x]	[ ]	MRI-based neuroimaging

Antibodies

Antibodies used	Synaptic Systems anti c-Fos, Catalogue# 226 003<br>Donkey-Anti-Rabbit AlexaFluor594 secondary antibody - Jackson Immunoresearch, Cat# 711-585-152<br>Donkey-Anti-Rabbit AlexaFluor790 secondary antibody - Invitrogen, Cat# A11374
Validation	This antibody has been validated via western blot, showing a dense band at the correct molecular weight for c-Fos, as well as the

predicted nuclear expression pattern via immunohistochemistry.

Animals and other organisms

Policy information about studies involving animals; ARRIVE guidelines recommended for reporting animal research

Laboratory animals	We used male Long Evans rats weighing 275–300 g (i.e. 2-3 months age), purchased from Charles River Laboratories. We also used male C57Bl6/J mice aged 2-3 months, purchased from Jackson Laboratories. Transgenic strains include male D1-Cre, D2-Cre, and Chat-Cre mice congenic on a C57Bl6/J background, 2-3 months age bred within our laboratory. Heterozygous offspring from FosCreERT2 & Fos2A-iCreER mice obtained from Jackson Laboratories bred with C57Bl6/J mice for one generation, 2-3 months of age were also used. Animals were housed at ambient temperatures of 65-75°F with 40-60% humidity.
Wild animals	No wild animals were used in these studies.
Field-collected samples	No field-collected samples were used in these studies.
Ethics oversight	All studies were approved by the Institutional Animal Care & Use Committee at the Icahn School of Medicine at Mount Sinai and the Scripps Research Institute.

Note that full information on the approval of the study protocol must also be provided in the manuscript.
